# Genetic Variants Contributing to Colistin Cytotoxicity: Identification of *TGIF1* and *HOXD10* Using a Population Genomics Approach

**DOI:** 10.3390/ijms18030661

**Published:** 2017-03-18

**Authors:** Michael T. Eadon, Ronald J. Hause, Amy L. Stark, Ying-Hua Cheng, Heather E. Wheeler, Kimberly S. Burgess, Eric A. Benson, Patrick N. Cunningham, Robert L. Bacallao, Pierre C. Dagher, Todd C. Skaar, M. Eileen Dolan

**Affiliations:** 1 Division of Nephrology, Indiana University School of Medicine, Indianapolis, IN 46202, USA; yicheng@iu.edu (Y.-H.C.); rbacalla@iu.edu (R.L.B.); pdaghe2@iu.edu (P.C.D.); 2 Division of Clinical Pharmacology, Indiana University School of Medicine, Indianapolis, IN 46202, USA; ksburges@iu.edu (K.S.B.); eabenson@iu.edu (E.A.B.); tskaar@iu.edu (T.C.S.); 3 Juno Therapeutics, Seattle, WA 98109, USA; ronaldhause@junotherapeutics.com; 4 Department of Biological Sciences, University of Notre Dame, South Bend, IN 46556, USA; astark1@nd.edu; 5 Department of Biology, Department of Computer Science, Loyola University Chicago, Chicago, IL 60660, USA; Hwheeler1@luc.edu; 6 Department of Medicine, University of Chicago, Chicago, IL 60637, USA; pcunning@medicine.bsd.uchicago.edu

**Keywords:** colistin, lymphoblastoid cell line, nephrotoxicity, *TGIF1*, *HOXD10*

## Abstract

Colistin sulfate (polymixin E) is an antibiotic prescribed with increasing frequency for severe Gram-negative bacterial infections. As nephrotoxicity is a common side effect, the discovery of pharmacogenomic markers associated with toxicity would benefit the utility of this drug. Our objective was to identify genetic markers of colistin cytotoxicity that were also associated with expression of key proteins using an unbiased, whole genome approach and further evaluate the functional significance in renal cell lines. To this end, we employed International HapMap lymphoblastoid cell lines (LCLs) of Yoruban ancestry with known genetic information to perform a genome-wide association study (GWAS) with cellular sensitivity to colistin. Further association studies revealed that single nucleotide polymorphisms (SNPs) associated with gene expression and protein expression were significantly enriched in SNPs associated with cytotoxicity (*p* ≤ 0.001 for gene and *p* = 0.015 for protein expression). The most highly associated SNP, chr18:3417240 (*p* = 6.49 × 10^−8^), was nominally a *cis*-expression quantitative trait locus (eQTL) of the gene *TGIF1* (transforming growth factor β (TGFβ)-induced factor-1; *p* = 0.021) and was associated with expression of the protein HOXD10 (homeobox protein D10; *p* = 7.17 × 10^−5^). To demonstrate functional relevance in a murine colistin nephrotoxicity model, HOXD10 immunohistochemistry revealed upregulated protein expression independent of mRNA expression in response to colistin administration. Knockdown of *TGIF1* resulted in decreased protein expression of HOXD10 and increased resistance to colistin cytotoxicity. Furthermore, knockdown of *HOXD10* in renal cells also resulted in increased resistance to colistin cytotoxicity, supporting the physiological relevance of the initial genomic associations.

## 1. Introduction

Colistin (polymixin E) is a cyclic polypeptide antibiotic prescribed to treat resistant Gram-negative infections [1,2]. In clinical settings, the drug is administered as an anionic prodrug, colistin methanosulfate, which is subsequently hydrolyzed to the cationic colistin [3]. The major toxicities of colistin include nephrotoxicity [4] and neurotoxicity [5]. The use of colistin is often limited by nephrotoxicity, affecting up to 55% of recipients [6,7,8,9,10]. The deleterious consequences of nephrotoxicity include increased morbidity, mortality, and the development of chronic kidney disease. Clinical predictors of colistin nephrotoxicity do exist [11,12]; however, no genetic predictors of colistin nephrotoxicity have been discovered. The utility of the drug would be enhanced by the discovery of pharmacogenomic markers associated with toxicity that could be used to predict patients requiring dose reduction, altered dosing intervals, or increased monitoring to prevent nephrotoxicity.

Lymphoblastoid cell lines (LCLs) are a human cell-based model system that has been successfully utilized to identify genomic markers associated with cellular sensitivity to chemotherapeutics [13,14,15,16,17,18] and statins [19,20]. For this purpose, the LCL model from the International HapMap Consortium [21] has several advantages including publically available genetic information [21], gene expression [22], and protein expression data [23] to correlate with cytotoxicity (cell growth inhibition following drug exposure) phenotypes. Furthermore, associations between genetic variants and drug responses in LCLs have been replicated in patient samples [24,25,26,27]. Although tissue-specific patterns of gene and protein expression exist [28,29], the LCL model of drug-induced cytotoxicity captures components of the underlying polygenic architecture in non-hematologic toxicities of patients [30]. For example, single nucleotide polymorphisms (SNPs) associated with capecitabine-induced LCL cytotoxicity were enriched among SNPs associated with patient phenotypes of hand-foot syndrome [31]. Paclitaxel cytotoxicity-associated SNPs were likewise enriched among SNPs identified in a clinical trial of paclitaxel sensory peripheral neuropathy [32]. These successful translations from the LCL model to patients form the basis of our use of the LCL model to uncover genetic predictors of colistin toxicity.

In this report, we utilize an unbiased, comprehensive approach to identify genetic variants associated with colistin cytotoxicity that were further evaluated for potential functional significance through their association with gene expression and protein expression. The most highly associated SNP, located at chr18:3417240, was associated with colistin cytotoxicity and was a nominal *cis*-expression quantitative trait locus (eQTL) of its host gene, *TGIF1* (transforming growth factor β (TGFβ)-induced factor-1), a homeobox gene and negative regulator of TGF-β [33,34]. The SNP was similarly associated with the expression of the multimeric form of a protein, HOXD10 (homeobox protein D10). To validate the associations from our population genetic analyses and establish the relevance of these genetic associations in renal tissue, small interfering RNA (siRNA) knockdowns of *TGIF1* and *HOXD10* were undertaken in renal proximal tubular epithelial cells [35], among the cells most susceptible to renal toxicity [36]. 

The pharmacogenomic markers identified through these studies reveal important considerations in the molecular biology and pathogenesis of colistin toxicity and can be evaluated as predictors of toxicity in future clinical settings. 

## 2. Results

### 2.1. Colistin-Induced Cytotoxicity in Lymphoblastoid Cell Lines

Lymphoblastoid cell lines derived from individuals within the Yoruban (YRI) YRI1 and YRI3 populations were assessed for sensitivity to colistin. The mean half maximal inhibitory concentration (IC_50_) for all 68 unrelated cell lines was 176.5 *±* 6.6 µM. Phenotypes were generated from the log_2_ IC_50_ of each cell line and a histogram of the distribution of these phenotypes is illustrated in Figure S1. These phenotypes were normally distributed, passing a Kolmogorov–Smirnov normality test (*p* ≥ 0.05).

### 2.2. Genome-Wide Association Study of Colistin Cytotoxicity

**Figure 1:**
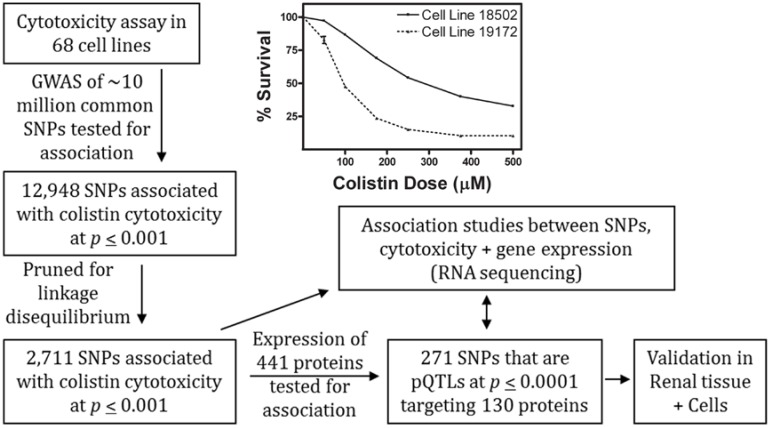
Schematic diagram of experimentation and analyses. Association studies were performed in 68 Yoruban (YRI) cells using 10 million single nucleotide polymorphisms (SNPs) (imputed), baseline gene expression measured by RNA sequencing (RNAseq), and baseline protein expression measured by microwestern and reverse phase protein arrays. Given the pertinence of colistin toxicity to human kidney injury, associations were then validated in human renal proximal tubular cells. GWAS: Genome-wide association study; pQTLs: Protein quantitative trait loci.

**Figure 2:**
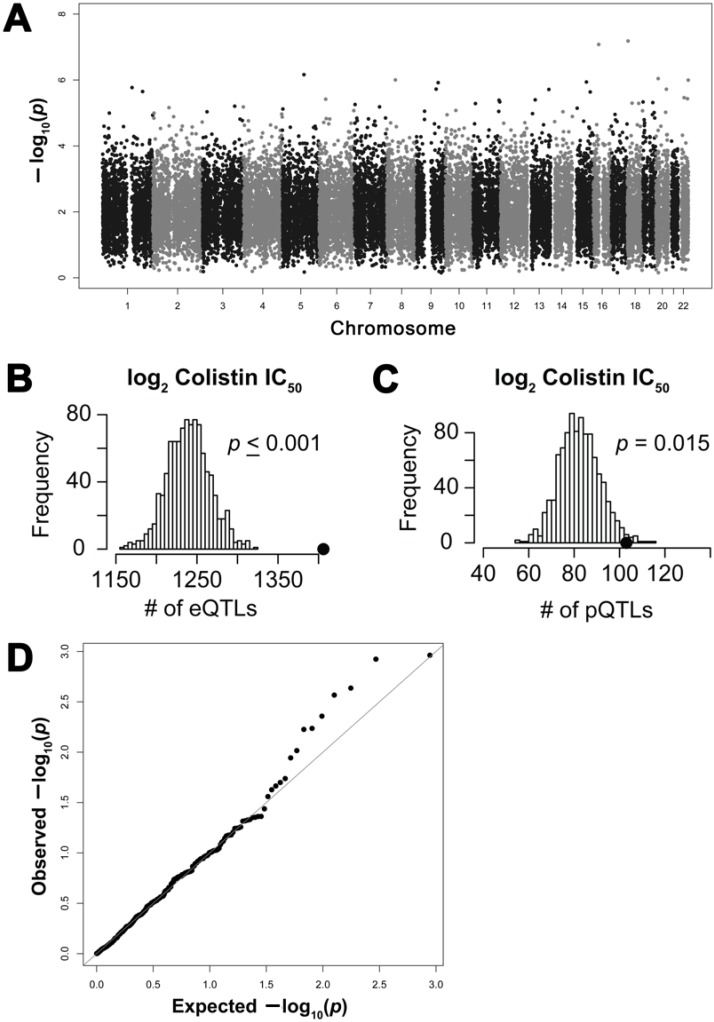
Genetic association studies: (**A**) Manhattan plot of association between SNPs and colistin half maximal inhibitory concentration (IC_50_). At *p* ≤ 0.001^3^, 12,948 SNPs (2,711 after Linkage Disequilibrium correction) were associated with cytotoxicity. The top SNP was located on chromosome 18, associated at *p* = 6.49 × 10^−8^. (**B**) Global enrichment analysis of the distribution of expression quantitative trait locus (eQTL) counts in 1000 simulations, each matching the Minor Allele Frequency distribution of all colistin-associated SNPs at *p* ≤ 0.001 (after LD correction). The black dot (●) represents the observed eQTL count (*n* = 1402 eQTLs at *p* ≤ 0.0001) in the colistin susceptibility-associated SNPs. Colistin-associated SNPs are enriched for eQTLs (*p* ≤ 0.001). (**C**) The pQTL enrichment analysis of the 441 proteins quantified by microwestern and reverse phase protein arrays. Colistin-associated SNPs at *p* ≤ 0.001 are enriched for mRNA-independent protein quantitative trait loci (pQTLs) among these (*n* = 271 pQTLs at *p* ≤ 0.0001). The black dot (●) represents the observed pQTL count (*n* = 104) in the colistin susceptibility-associated SNPs (*p* = 0.015). (**D**) Quantile*–*quantile (Q–Q) plot representing the observed associations between baseline protein expression and colistin IC_50_. At *p* ≤ 0.05, 23 proteins were significantly associated. Solid line indicates a false discovery rate (FDR) of 0.05.

Figure 1 illustrates our overall approach. A genome-wide association study (GWAS) performed with colistin log_2_ IC_50_ phenotypes did not result in any SNPs meeting Bonferroni genome-wide significance at *p* ≤ 5 × 10^−8^ (Figure 2A); however, 12,948 SNPs were associated with the log_2_ IC_50_ of colistin cytotoxicity at a nominal significance threshold of *p* ≤ 0.001. After pruning for linkage disequilibrium (LD), 2711 SNPs in separate recombination blocks were significant at this threshold. These SNPs were defined as drug quantitative trait loci (dQTLs) and are listed in Table S1.

### 2.3. Functional Enrichment of Expression Quantitative Trait Loci and Protein Quantitative Trait Loci

To determine whether colistin cytotoxicity dQTLs were enriched in SNPs that were also associated with gene expression as has been observed for several chemotherapeutics [37], we performed a permutation analysis with eQTLs defined as those SNPs associated with at least one of 18,227 genes measured by RNA sequencing (RNAseq) at a threshold of *p* ≤ 0.0001 [38]. From among the 2711 dQTLs, 1402 SNPs were also eQTLs and were significantly enriched when compared to that expected by random chance (Figure 2B, empirical *p* ≤ 0.001). 

Using a protein dataset composed of 441 human signaling and transcription factor proteins that were quantified in these cell lines using a microwestern and/or reverse phase protein array [23,39], we evaluated for the enrichment of protein quantitative trait loci (pQTLs); genotype correlated to protein expression at *p* ≤ 0.0001). Of the 2711 dQTLs, 271 were associated with baseline expression of 130 unique proteins (Supplementary Table S2). However, mRNA expression significantly affected expression of many of these protein associations. After correcting for mRNA expression, 104 SNPs were identified as pQTLs independent of eQTLs. The 104 mRNA-independent pQTLs represent a significantly enriched functional set of SNPs (Figure 2C, *p* = 0.015). 

### 2.4. Association of Protein Expression with Colistin Cytotoxicity

The direct correlation between baseline protein expression of the 441 proteins and colistin log_2_ IC_50_ was then queried. The significance of these associations is provided in a quantile–quantile (Q–Q) plot with a multiple testing corrected false discovery rate (FDR) of 0.05 (Figure 2D). Fourteen proteins had observed associations stronger than those expected by chance. At a relaxed *p* ≤ 0.05, 23 proteins were associated with colistin cytotoxicity (Table S3).

### 2.5. Evaluation of the Most Significant Single Nucleotide Polymorphism Associated with Colistin Cytotoxicity

**Figure 3:**
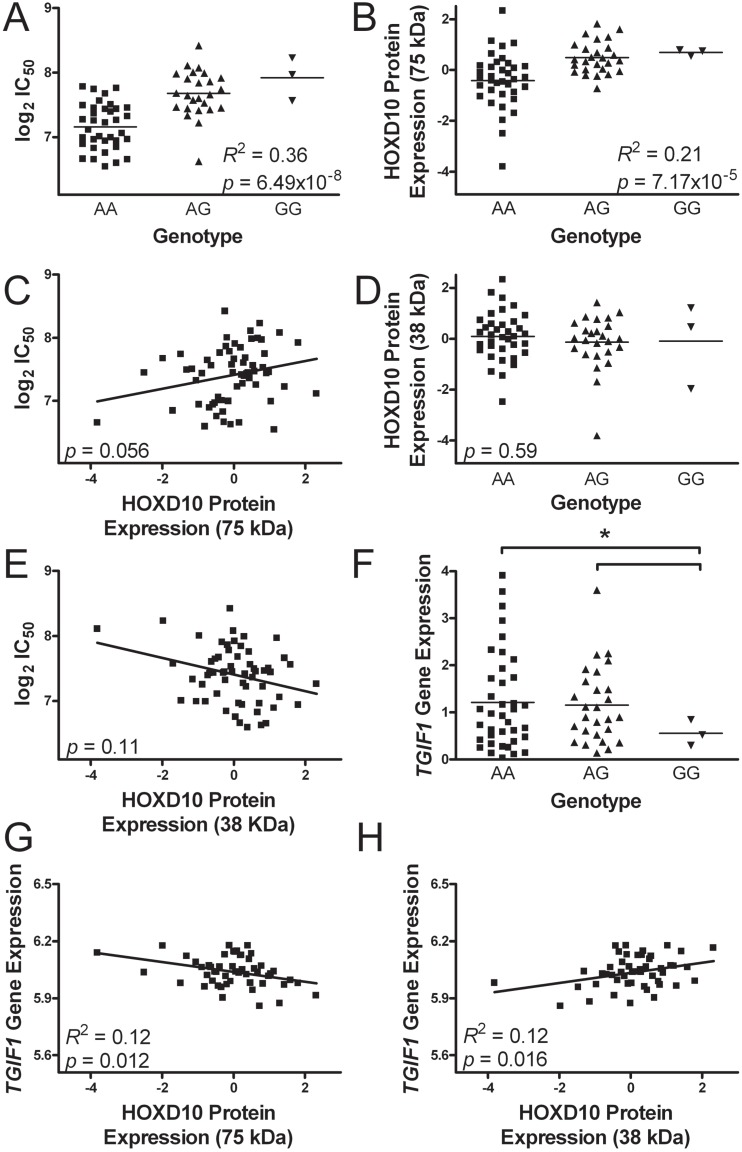
Association studies of chr18:3417240, *TGIF1* (transforming growth factor β (TGFβ)-induced factor-1) and the protein HOXD10 (homeobox protein D10): (**A**) The SNP chr18:3417240 is associated with colistin cytotoxicity (log_2_ IC_50_) at *p* = 6.47 × 10^−8^; (**B**) Chr18:3417240 was associated with expression of HOXD10 (75 kDa isoform) at *p* = 7.17 × 10^−5^; (**C**) HOXD10 protein expression (75 kDa) was not significantly associated with colistin cytotoxicity at *p* = 0.056; however, the direction of effect for this result is consistent with A and B; (**D**) Chr18:3417240 was not associated with expression of HOXD10 (38 kDa isoform) at *p* = 0.59; (**E**) HOXD10 protein expression (38 kDa) was not significantly associated with colistin cytotoxicity at *p* = 0.11; (**F**) the homozygous recessive (GG) genotype of chr18:3417240 had significantly lower *TGIF1* gene expression than heterozygous (AG, *p* = 0.031) or homozygous dominant (AA, *p* = 0.021) lymphoblastoid cell lines; (**G**) *TGIF1* gene expression is associated with HOXD10 protein expression (75 kDa) at *p* = 0.012 (from Illumina array); and (**H**) *TGIF1* gene expression is associated with HOXD10 protein expression (38 kDa) at *p* = 0.016 (from Illumina array). The association between *TGIF1* gene expression and either 75 kDa or 38 kDa HOXD10 protein expression was not significant as measured by the RNA sequencing platform (see Figure S2).

The SNP most significantly associated with colistin cytotoxicity in the 68 cell lines was an imputed intronic SNP located on chromosome 18 at base pair 3,417,240 (GRCh38/hg38) in the gene *TGIF1*. The genotype of this dQTL, hereby referred to as chr18:3417240, was correlated with the log_2_ IC_50_ for colistin at a level close to the genome-wide significance threshold of 5 × 10^−8^ (*R*^2^ = 0.36, *p* = 6.49 × 10^−8^, Figure 3A). The minor allele (G) frequency was 24.3% in the population studied and this allele conferred resistance to colistin cytotoxicity. Although the association between chr18:3417240 and colistin cytotoxicity nearly reached a genome-wide threshold of significance, many downstream associations with baseline protein and baseline gene expression in LCLs did not approach Bonferroni-corrected levels of significance. We present these downstream targets to glean insight into the functional relationship between the SNP and colistin cytotoxicity.

### 2.6. chr18:3417240 and Homeobox Protein D10 Expression

To better understand how chr18:3417240 might mediate resistance to colistin cytotoxicity, we examined whether it was also an eQTL or pQTL. In both the genotype-RNAseq expression and genotype-protein association studies, chr18:3417240 was most strongly associated with expression of the protein HOXD10. Homeobox protein D10 is encoded by its gene located on chromosome 2. The monomeric form is 38 kDa in size, but it is well known to form multimers by dimerization, heterodimerization, or trimerization [40,41]. On the microwestern array, both a 38 kDa HOXD10 isoform and a 75 kDa isoform were found. It is possible that the 75 kDa isoform represents a multimeric or cross-hybridized form of HOXD10; however, the sensitivity of the microwestern array is insufficient to discern the exact size and nature of this isoform.

Chr18:3417240 was identified as a *trans*-pQTL, associated with expression of the 75 kDa form of the protein HOXD10 (*R*^2^ = 0.21, *p* = 7.17 × 10^−5^). The G allele correlated with higher levels of 75 kDa protein expression on the microwestern array (Figure 3B). Based on the positive correlation of the G allele with both 75 kDa expression and the log_2_ IC_50_ of colistin (from Figure 3A,B), the 75 kDa expression is predicted to correlate positively with the log_2_ IC_50_ of colistin (i.e., greater HOXD10 75 kDa expression predicts resistance to colistin cytotoxicity). While the directionality of this association followed predictions, the relationship between 75 kDa protein expression and the log_2_ IC_50_ of colistin failed to reach significance (*p* = 0.056, Figure 3C).

A second HOXD10 isoform of approximately 38 kDa was also detected on the microwestern array. This 38 kDa isoform may represent the monomeric form of HOXD10 on the microwestern array. The genotype of chr18:3417240 was not significantly associated with expression of 38 kDa HOXD10 (Figure 3D). Further, expression of the 38 kDa HOXD10 did not significantly correlate with the log_2_ IC_50_ of colistin (*p* = 0.11, Figure 3E). Although non-significant, both relationships held the opposite direction of effect to the 75 kDa HOXD10 protein expression (i.e., reduced 38 kDa HOXD10 predicts resistance to colistin).

We then examined whether *HOXD10* baseline gene expression by RNAseq correlated with genotype, protein expression, or cytotoxicity. Unfortunately, *HOXD10* gene expression was only detected above background thresholds in 3 of 68 cell lines. Thus, no relationship with gene expression could be established using the RNAseq expression [38]. We entertained the idea that *HOXD10* gene expression was insufficiently captured by RNAseq and examined baseline LCL expression on a second platform, an Illumina microarray [42]. Although expression measurements were available for 53 of the LCLs, the dynamic range of expression was small. No significant associations between *HOXD10* gene expression and genotype, protein expression, or cytotoxicity were found. Thus, chr18:3417240 appears to be an mRNA-independent pQTL of *HOXD10*. However, we cannot exclude the possibility that small, undetectable differences in gene expression mediate the variation in protein expression.

### 2.7. Transforming Growth Factor β-Induced Factor-1 Gene Expression

Chr18:3417240 genotype was associated with expression of its host gene *TGIF1* (Figure 3F). Using gene expression measured by RNAseq, the homozygous recessive genotype (GG) was associated with lower expression of *TGIF1* as compared to either the homozygous dominant (AA, *p* = 0.021) or heterozygous (*p* = 0.031) genotypes. No significant expression difference was observed between the AA and AG genotypes.

We then sought to explore the relationship between *TGIF1* gene expression with both colistin cytotoxicity and HOXD10 protein expression. Based on the correlation of the GG genotype with increased log_2_ IC_50_ of colistin, increased 75 kDa HOXD10 expression, and reduced *TGIF1* expression (from Figure 3A,B,F), higher *TGIF1* gene expression is predicted to: (i) negatively correlate with the log_2_ IC_50_ of colistin (i.e., knockdown of gene expression would increase cellular sensitivity to colistin); (ii) negatively correlate with 75 kDa expression of HOXD10; and (iii) positively correlate with 38 kDa HOXD10 expression. None of these three associations reached statistical significance using expression data from RNAseq (Figure S2). 

To complement the RNAseq expression data, we again examined a second set of expression data for the same LCLs from the Illumina array platform. Using these expression data, *TGIF1* gene expression did correlate significantly with HOXD10 protein expression (Figure 3G–H). Consistent with the predictions above, TGIF1 expression negatively correlated with 75 kDa HOXD10 expression (*R*^2^ = 0.123, *p* = 0.012) and positively with 38 kDa HOXD10 expression (*R*^2^ = 0.12, *p* = 0.016). The direction of effect for the Illumina array associations was the same as those of the RNAseq associations. The association between the GG genotype of chr18:3417240 and *TGIF1* expression on the Illumina array persisted (Figure S2, *p* = 0.05), in accordance with the results from RNAseq. The relationship between *TGIF1* expression and log_2_ IC_50_ of colistin remained non-significant on the Illumina platform. These data support the hypothesis that chr18:3417240 impacts *TGIF1* expression and that *TGIF1* expression in turn affects HOXD10 protein isoform expression.

### 2.8. Homeobox Protein D10 Expression Is Upregulated in the Kidney in Response to Colistin, Independent of Its Gene Expression

One goal of this investigation was to identify genetic variants in LCLs that predict susceptibility to colistin-induced nephrotoxicity; however, gene and protein expression patterns vary in different tissues [28,29]. Variants, genes, and proteins discovered in LCLs may not be associated with colistin toxicity in the kidney. As a result, we sought to determine whether the HOXD10 protein was expressed in kidney and whether this expression was affected by colistin administration.

**Figure 4:**
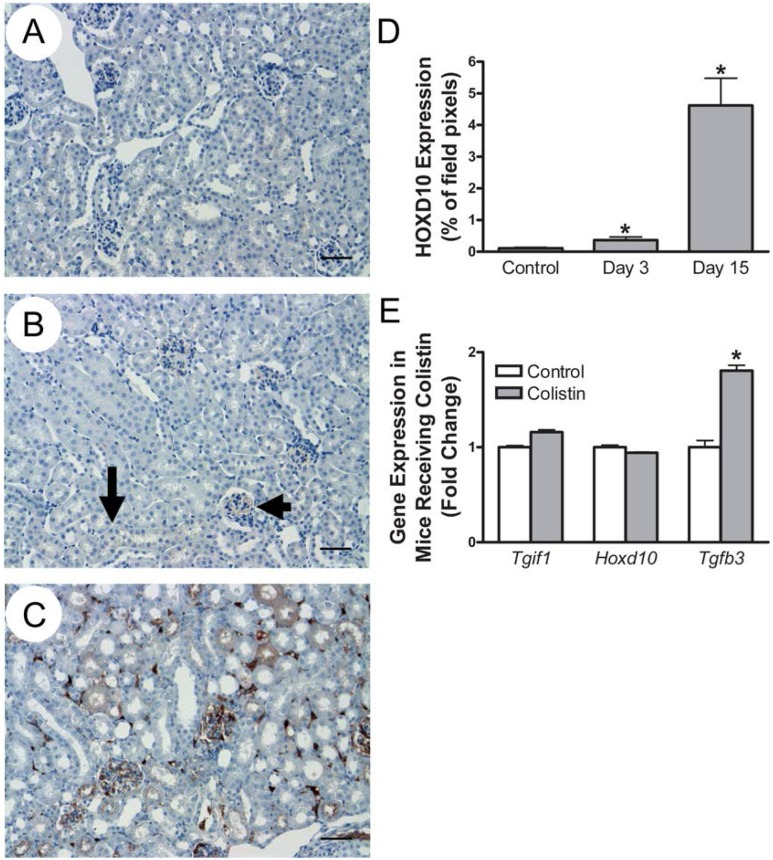
Homeobox protein D10 expression is increased in mouse kidney following colistin exposure: (**A**) HOXD10 immunohistochemistry (IHC) in control mice revealed minimal staining. (**B**) HOXD10 IHC staining is upregulated in mice after three days of colistin exposure. Subtle, but significant up-regulation of expression is noted in glomeruli (arrowhead) and the brush border of proximal tubules (arrow). (**C**) HOXD10 IHC is upregulated after 15 days of colistin exposure. Glomeruli, interstitium, S1–S2 proximal tubular brush borders, and S3 proximal tubules were affected. Scale bar = 50 µm. Magnification: 20×. (**D**) Quantitation of HOXD10 protein expression by IHC reveals increased expression on Day 3 and Day 15 of colistin administration. (**E**) *TGFB3* (transforming growth factor β-3) gene expression was upregulated in mouse kidney tissue. * = *p* ≤ 0.05.

Mice were administered either colistin (16 mg/day) or saline intraperitoneally and sacrificed on day 3 or 15 as previously described [36]. The dose was approximately twice the upper limit of the recommended corresponding weight-based human dose and the timing reflected a typical two-week course of antibiotics. Homeobox protein D10 expression and distribution was assessed in formalin fixed kidney by immunohistochemistry (IHC; Figure 4A–C). Even before the onset of overt pathologic kidney injury, HOXD10 protein expression was subtly but significantly upregulated after three days of colistin exposure (Figure 4D, 0.368% *±* 0.1%) compared with control (0.106% *±* 0.03%, *p* = 0.044), as determined by the total proportion of stained pixels in a 40× field. HOXD10 was more remarkably upregulated after 15 days of colistin administration (4.62% *±* 0.86%, *p* = 0.00079). Multiple portions of the kidney were affected, including the glomeruli, interstitium, and proximal tubules. S1 and S2 proximal tubules revealed increased staining in the luminal brush border. S3 proximal tubules of the outer medullary stripe revealed upregulated cytoplasmic staining with some nuclear staining. These data provide the rationale for the functional validation of the *TGIF1*–HOXD10 axis in normal human proximal tubular kidney (NHPTK) cells below. 

To understand whether gene expression played a role in the HOXD10 protein expression changes, we extracted data from an Illumina Mouse WG-6 Expression array performed in control mice and those receiving colistin for three days (Figure 4E). Transforming growth factor β is known to interact with *Tgif1*. *Tgfb3* (transforming growth factor β-3) gene expression was significantly upregulated (1.81 *±* 0.058 fold increase, *p* = 0.016), suggesting this pathway is activated in mice receiving colistin. *Hoxd10* gene expression was not significantly changed (94% *±* 0.8% of control, *p* = 0.699). This may be consistent with the lack of mRNA and protein expression correlation observed in LCLs; however, small changes in gene expression at the limits of detection cannot be excluded. *Tgif1* gene expression was not significantly increased (1.16 *±* 0.02-fold increase, *p* = 0.11). Speculation regarding the lack of significant differential *Tgif1* expression after three days of colistin is provided in the discussion. The murine kidney IHC and array data presented here provided us enough justification to move forward with the functional validation outlined below. These data will also provide the basis for future gene knockdown experimentation and pathway interrogation in organisms.

### 2.9. Colistin-Induced Cytotoxicity in Human Proximal Tubular Cells

**Figure 5:**
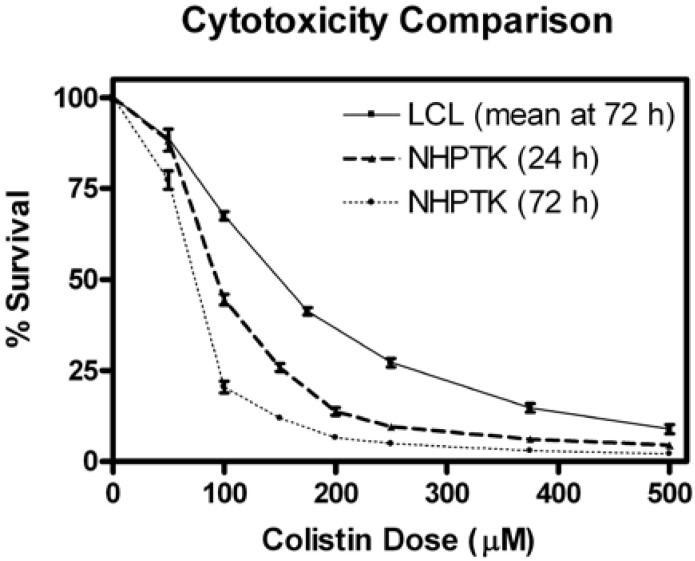
Cytotoxicity in LCLs and normal human proximal tubular kidney (NHPTK) cells. A mean of all 68 cytotoxicity curves is provided for the LCLs 72 h after colistin exposure. Normal human proximal tubular kidney cells were more sensitive to colistin than LCLs at 72 h. Normal human proximal tubular kidney cells exposed to colistin had relatively greater survival at 24 h as compared to 72 h.

The cellular sensitivity of NHPTK cells to colistin was assessed 24 and 72 h after exposure. Normal human proximal tubular kidney cells are primary renal tubular epithelial cells from a single donor that have been immortalized by human telomerase elongation. When maintained in renal epithelial growth medium, the NHPTK cell line closely resembles primary proximal tubular cells with a renal phenotype that includes transporter expression and function, tight junction formation, and parathyroid hormone responsiveness [35,43]. Normal human proximal tubular kidney cells were more sensitive to colistin than LCLs (Figure 5). This finding parallels the clinical side effect profile of colistin which includes nephrotoxicity, but not hematotoxicity. The mean IC_50_ in LCLs and NHPTK cells were 176.5 *±* 6.6 µM and 65 *±* 6.0 µM after 72 h of drug exposure, respectively. The IC_50_ in NHPTK cells after 24 h was 94.7 *±* 5.9 µM. Given the susceptibility of NHPTK cells to colistin, the 24 h time point was selected for subsequent analyses.

### 2.10. Smal Interfering RNA Knockdown of Transforming Growth Factor β-Induced Factor-1 and Homeobox Protein D10 Alters Gene and Protein Expression in Renal Cells

**Figure 6:**
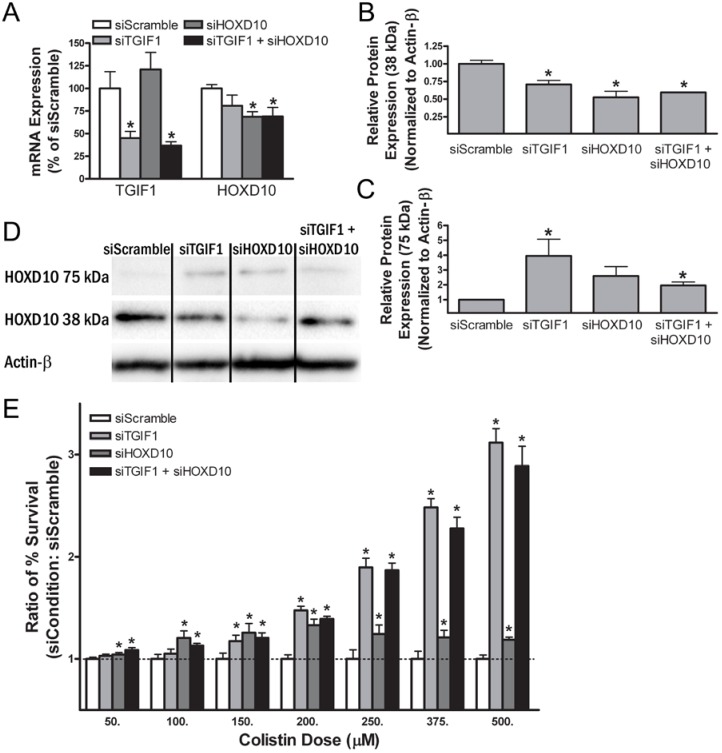
Effect of gene knockdown on gene expression, protein expression, and cellular resistance to colistin. Following transfection with small interfering RNA (siRNA), the effect on gene expression (**A**); protein expression of HOXD10 (**B**–**D**); and colistin-induced cytotoxicity (**E**) was determined for a control scramble siRNA (siScramble or siRNA targeting TGIF1, HOXD10, and the combination of both. was determined for: Knockdown was performed with at least three biological replicates (six or more technical replicates). mRNA levels were measured by real-time quantitative polymerase chain reaction (qRT-PCR) 24 h post-siRNA transfection. Gene expression is given as a percentage of expression relative to siScramble after Glyceraldehyde 3-phosphate dehydrogenase (GAPDH) normalization. Protein expression was calculated from densitometry with an Actin-β control and then compared to siScramble. Cytotoxicity was measured 24 h after initial colistin exposure. Cell survival at each concentration was calculated relative to control (no drug). The relative increase in % cell survival is expressed as a ratio of each condition’s knockdown (siCondition) to the siScramble. * = *p* ≤ 0.05 by Student’s *t*-test between the scramble siRNA and the siRNA targeting each gene. Baseline HOXD10 gene expression was low in NHPTK cells, contributing to the modest knockdown effect.

To validate the functional significance of *TGIF1* gene expression and HOXD10 protein expression in mediating colistin cytotoxicity, mRNA expression of TGIF1 and HOXD10 were reduced with siRNA knockdown and cellular sensitivity was compared to a scrambled siRNA (siScramble) control. Four conditions were evaluated: siScramble control, siTGIF1, siHOXD10, and siTGIF1 + siHOXD10 (a combined knockdown). siRNA knockdown of TGIF1 reduced its gene expression to 45.1% *±* 7.5% 24 h after knockdown compared to the time matched siScramble control (*p* = 0.018, Figure 6A). siHOXD10 displayed no significant effect on expression of TGIF1 (1.21 fold increase, *p* = 0.47). The combination of siTGIF1 + siHOXD10 knockdown reduced TGIF1 expression to 36.8% *±* 1.9% (*p* = 0.027) as compared to siScramble 24 h after knockdown.

In the population genomic studies described above, HOXD10 protein expression did not significantly correlate with *HOXD10* gene expression. Despite this, we posited that siRNA knockdown of HOXD10 mRNA might still result in a significant change in HOXD10 protein expression. Based on the associations in Figure 3G–H, we further hypothesized that *TGIF1* knockdown would reduce monomeric HOXD10 protein expression and increase 75 kDa HOXD10 protein expression. 

Homeobox protein D10 was lowly expressed by real-time quantitative polymerse chain reaction (qRT-PCR) in NHPTK cells with raw threshold cycle (C_T_) values in the 30–32 range. By contrast, TGIF1 C_T_ values were approximately 21–22. Nonetheless, HOXD10 expression was modestly reduced to 68.7% *±* 5.6% of the siScramble control 24 h after knockdown (*p* = 0.0068). Homeobox protein D10 expression was reduced to 69.1% *±* 9.9% of the control (*p* = 0.023) after the combined siTGIF1 + siHOXD10 knockdown. siTGIF1 did not cause a significant reduction in *HOXD10* gene expression (80.8% *±* 11.6%, *p* = 0.18).

Homeobox protein D10 expression of both the 38 kDa and 75 kDa isoforms was assessed by Western immunoblot for each of the four conditions after siRNA knockdown (*n* = 5 for each condition). siHOXD10 resulted in a reduction of its own monomeric protein expression (52.6% *±* 8.7% of control, *p* = 0.0022) as estimated by densitometry (Figure 6B–D). Despite the lack of *HOXD10* gene and protein expression correlation in the population genomics studies, this finding is consistent with the conclusion that relatively modest reductions in HOXD10 mRNA can result in reductions in its protein expression. siTGIF1 + siHOXD10 resulted in a similar reduction in monomeric HOXD10 protein expression (59.4% *±* 0.5% of control, *p* = 0.0009) as estimated by densitometry. Finally, siTGIF1 knockdown also resulted in a reduction in monomeric HOXD10 protein expression (70.7% *±* 5.9% of control, *p* = 0.0087) as estimated by densitometry. 

In contrast, the siTGIF1 and siTGIF1 + siHOXD10 conditions showed increased levels of protein detected by the HOXD10 antibody at 75 kDa (siTGIF1: 3.5 *±* 1.4-fold increase, *p* = 0.044; siTGIF1 + siHOXD10: 1.5 *±* 0.2-fold increase, *p* = 0.040). siHOXD10 did not significantly increase 75 kDa HOXD10 (2.2 *±* 0.6-fold, *p* = 0.065). In NHPTK cells, total HOXD10 protein was still reduced in all conditions because the proportion of the 38 kDa protein far outweighed the 75 kDa protein. 

These findings support the population genomics results presented in Figure 3. Expression of the *TGIF1* gene was positively correlated with monomeric HOXD10 protein expression and negatively correlated with 75 kDa HOXD10 expression. Given the limitations in determining *HOXD10* gene expression by RNAseq, Illumina array, and qRT-PCR, it cannot be determined from these studies whether *TGIF1* gene expression changes result in HOXD10 protein changes via downstream post-translational modifications or by subtle reductions in gene expression.

### 2.11. Small Interfering RNA Knockdown of Transforming Growth Factor β-Induced Factor-1 and Homeobox Protein D10 Alters Sensitivity to Colistin in Normal Human Proximal Tubular Kidney Cells

The effect of siRNA knockdown of TGIF1 and HOXD10 mRNA on colistin cytotoxicity was assessed in NHPTK cells. Based on the population genomic studies presented in Figure 3 and Figure S2, a higher colistin log_2_ IC_50_ (i.e., increased resistance to colistin cytotoxicity) correlated with the G allele of chr18:3417240. Increased resistance to colistin cytotoxicity is predicted to correlate with: (i) reduced *TGIF1* gene expression; (ii) reduced monomeric HOXD10 expression; and (iii) increased 75 kDa HOXD10 expression. Thus, siRNA knockdown of each condition (siTGIF1, siHOXD10, and siTGIF1 + siHOXD10) is expected to elicit an increase in resistance to colistin cytotoxicity as compared to a scrambled siRNA.

siTGIF1 knockdown resulted in an increase in cell survival in response to colistin (Figure 6E). Although no significant difference was observed at the 50 or 100 µM colistin dose, at higher doses of 150 µM to 500 µM, NHPTK cells were more resistant to colistin as compared to the dose matched siScramble. The relative increase (ratio of siTGIF1 to siScramble) for each concentration is provided in Figure 6E and ranged from a 17.4% to 211.8% increase in relative resistance. The absolute increase in resistance was modest but significant, ranging from 4.5% to 9.4% at each concentration of 150 µM or higher (*p* ≤ 0.05). Both the absolute and relative resistance increased with increasing doses.

siHOXD10 knockdown also resulted in an increase in cell survival in response to colistin as compared to the siScramble. Significant but modest increases were observed at each colistin dose. Compared with siTGIF1, the relative increase in resistance caused by siHOXD10 was more consistent across each colistin dose, ranging from 4.3% to 33.1% at each dose (*p* ≤ 0.05). The absolute increase in resistance ranged from 9.4% at the 100 µM dose down to only 0.8% at the 500 µM dose.

Combined mRNA reduction with siTGIF1 and siHOXD10 resulted in neither a synergistic nor additive effect; rather, the combined knockdown recapitulated the stronger of the effects from either siTGIF1 or siHOXD10 at each dose. For example, at lower colistin doses (50–150 µM), the relative and absolute increases in resistance were similar to siHOXD10 (8.7% to 20.7% relative increase, *p* ≤ 0.05). At higher concentrations (250–500 µM), the relative and absolute increases in resistance were similar to siTGIF1 (86.8% to 189.1% relative increase, *p* ≤ 0.05).

## 3. Discussion

In this investigation, the results of a comprehensive GWAS of colistin cytotoxicity are presented. Colistin cytotoxicity associated SNPs were enriched in eQTLs and mRNA-independent pQTLs. The data presented in this investigation suggest that chr18:3417240 is a dQTL of colistin, an mRNA independent pQTL of the protein HOXD10 and nominal *cis*-eQTL of its host gene *TGIF1*. After confirming in vivo kidney expression of HOXD10, these associations were then tested in human renal proximal tubular cells through siRNA knockdown, supporting the relationship of *TGIF1* and HOXD10. Specifically, the knockdown of *TGIF1* led to reduced protein expression of monomeric HOXD10. 

Transforming growth factor β-Induced homeobox 1, or *TGIF1*, is a protein-coding gene important in the TGF-β signaling axis [33]. In conjunction with Smad, it is a known repressor of TGF-β [34], and rises in response to elevated levels of the cytokine [44]. The TGIF1 protein binds directly to c-Jun to assemble with Smad proteins and mediate the transcriptional repression of TGF-β and other genes [45]. In the presented studies, TGIF1 siRNA knockdown resulted in increased cell survival following colistin exposure. Expression of TGIF1 has been demonstrated to regulate stem cell self-renewal [46] and in *TGIF1* (−/−) knockout mice, loss of expression correlated with higher repopulation of bone marrow cells [46]. The *TGIF1* gene is known to have over 20 splicing variants [47]. Transcript variant expression levels have been measured in tissues with over- or under-expression of certain variants correlating with the presence or absence of malignancy. In a study of oral squamous cell carcinomas, transcript TGIF1-008 was over-expressed and TGIF1-005 under-expressed in malignant tissue [48]. 

siTGIF1 resulted in reductions in HOXD10 protein expression in our studies. However, siTGIF1 and siHOXD10 had different patterns of cytotoxicity with siTGIF1 resulting in more pronounced changes at higher colistin doses. By contrast, siHOXD10 resulted in more consistent increases in cell survival across the dosing range. It is possible that mRNA reduction of TGIF1 resulted in a variety of downstream effects, including changes in expression of other genes and proteins resulting in both cell survival and cell death, with the balance shifting to cell survival only under higher concentrations of colistin. One potential limitation of the siRNA knockdown model is that a pool of siRNA constructs was used to knockdown all transcripts (as the exons near the 3’ end are conserved across transcript variants). Future studies should examine the role of transcript variant specific knockdown in cytotoxicity.

Homeobox D10, or HOXD10, is a 38 kDa protein with roles in regulation of the cell cycle and in the development of the spinal cord and kidney [49,50,51]. A mutation in *HOXD10* has been found to be a causative variant in Wilm’s tumor (a pediatric renal tumor) [52], further supporting its role in the kidney’s cell cycle. Homeobox protein D10 is a target of the microRNA miR-7, which suppresses p21/CDKN1a (Cyclin-dependent kinase inhibitor 1α) activated kinase [53]. Our group’s prior work has implicated G2/M cell-cycle arrest in the pathogenesis of colistin nephrotoxicity [36]. Thus, the role of HOXD10 in colistin cytotoxicity is plausible given its renal expression and link to the cell cycle. Although specific interactions between the *TGIF1* gene and Homeobox protein D10 have not been previously reported, these interactions are also plausible. Homeobox HOX proteins have DNA-binding sites, operating as transcription factors downstream of bone morphogenetic protein (BMP) signaling, part of the TGF-β signaling superfamily [54]. Smad1 and Smad6 interact directly through T-box 1 (Tbx1) with the HOXD10 protein, repressing its transcriptional activity [51]. Based on this, TGIF1 and HOXD10 likely interact through the TGF-β/BMP-signaling pathway, potentially linked by Smad proteins. 

Several limitations exist. First, this study identified genes in a lymphoblastoid cell model and replication in relevant cell types will be required to better elucidate downstream pathways. The 68 unrelated YRI samples are a relatively small sample size for GWAS. The association between chr18:3417240 and cytotoxicity, at *p* ≤ 6.49 × 10^−8^, failed to reach a genome-wide significance threshold of *p* ≤ 5 × 10^−8^. This limitation was addressed by the experimentation in NHPTK cells that supports the associations identified in the population genomics portion of the paper. Another limitation is that the pQTL association data is based on antibody-based, targeted proteomic array data [39] and the strength of the corresponding antibodies and methodology (including reducing conditions) used for quantification. Finally, other limitations include the polygenic nature of cytotoxicity and corresponding modest effect of any single gene knockdown, the need to overcome tissue specificity of expression, and the low gene expression of *HOXD10* on multiple platforms (RNAseq, Illumina, and qRT-PCR). 

Despite these limitations, this work identified two new genes that have not previously been considered in the pathogenesis of colistin nephrotoxicity. The genes *TGIF1* and *HOXD10* are rarely considered in the pathogenesis of acute kidney injury. Their identification in this study may result in better understanding of their roles in nephrotoxicity caused by a variety of drugs. Of note, neither *TGIF1* nor *HOXD10* were identified in a prior array based study of colistin nephrotoxicity in mice [36]. Since no population of genotyped renal cells exists to correlate with drug-related phenotypes, a strength of this investigation was the combination of the LCL model with renal tissue and cells to discover associations important in renal cytotoxicity. 

The ultimate goal is to use data such as that presented above to predict patients at risk for nephrotoxicity. To date, no prior GWAS studies have examined the role of genotype in predicting toxicity to colistin. Human GWAS studies should be performed; however, these will be fraught with at least one major obstacle: phenotype selection. In practice, clinicians still use creatinine as a late marker of acute kidney injury. This marker is burdened by confounders, and nephrotoxicity may prove one of multiple contributors to an episode of acute kidney injury. As new, more sensitive, biomarkers of acute kidney injury reach clinical practice, this difficulty may be attenuated. Despite remarkable differences in the IC_50_ of LCLs and renal cells to colistin, the genes and proteins identified in LCLs still resulted in alterations in renal cell sensitivity to colistin. Further, the clean phenotype of cytotoxicity allowed a small number of cell lines to be used to obtain important genetic information. Prospective pharmacogenomic implementation initiatives are underway [55,56]. Often, array platforms are used to screen a moderate number of variants important in drug dosing [57]. Future investigations could include the screening of chr18:3417240 in patients receiving colistin in order to improve the clinical validity of this SNP in predicting nephrotoxicity.

In conclusion, we present a comprehensive genome-wide association study of colistin mediated susceptibility. Through this investigation, we identified a SNP, chr18:3417240 strongly associated with colistin cytotoxicity. With additional validation, this SNP may serve as a marker for susceptibility to colistin in the future. Its host gene, *TGIF1*, and an associated protein HOXD10 were found to contribute to colistin cytotoxicity in renal proximal tubular cells. Both *TGIF1* and HOXD10 are worthy targets for future investigation of nephrotoxicity in humans. 

## 4. Materials and Methods

### 4.1. Cell Lines and Drug

Lymphoblastoid cell lines were cultured in RPMI 1640 media with 15% bovine growth serum (Hyclone, Logan, UT, USA) and 3.7 mM-glutamine. Cell lines were diluted three times per week to a concentration of 300,000–350,000 cells/mL and maintained in a 37 °C, 5% CO_2_ humidified incubator. Media and components were purchased from Cellgro (Herndon, VA, USA). HapMap LCLs were purchased from Coriell Institute for Medical Research (Camden, NJ, USA). The 68 LCLs of Yoruban ancestry (YRI 1 and YRI 3) were used in previous experiments evaluating protein levels [23,39]. Table S4 lists the cell lines.

Normal human proximal tubular kidney cells [35] were maintained in REGM media (Lonza, Basel, Switzerland) supplemented with 10% fetal bovine serum (HyClone). Normal human proximal tubular kidney cells were diluted to 20%–30% confluency three times a week and maintained at 37 °C in 95% humidified atmosphere with 5% CO_2_.

Colistin sulfate was commercially obtained from Sigma (St. Louis, MO, USA). 

### 4.2. Colistin-Induced Cytotoxicity

HapMap LCLs were phenotyped for cellular sensitivity to colistin using a short-term, colorimetric growth inhibition assay. Cell lines were maintained at exponential growth phase with ≥85% viability as determined using the Vi-Cell XR viability analyzer (Beckman Coulter, Fullerton, CA, USA) and diluted in triplicate at a density of 10^5^ cells/mL in 96-well round-bottom plates (Corning, Inc., Corning, NY, USA) 24 h prior to drug treatment. Colistin was prepared by dissolving powder in sterile water to obtain a stock solution of 25 mM with subsequent drug filtration. The drug was dissolved in water rather than saline or phosphate-buffered saline (PBS) because of its increased stability [58]. The drug was suspended in media and in turn added to the 96-well plates of each cell line to obtain six final concentrations of colistin (50, 100, 175, 250, 375 and 500 µM). The range of concentrations was carefully chosen based on previously published in vitro cytotoxicity data [59,60] and optimized for these cell lines. Alamar Blue (Life Technologies, Carlsbad, CA, USA) was added 48 h after drug addition and 24 h before absorbance reading at wavelengths of 570 and 600 nm using the Synergy-HT multi-detection plate reader (BioTek, Winooski, VT, USA). Percent survival was quantified relative to a control well without drug addition. Experiments consisted of at least two biological replicates and six technical replicates. The phenotype chosen for analysis was the IC_50_ calculated from the survival curve.

Since NHPTK cells are adherent, they were plated at a density of 2 × 10^4^ in 96-well flat-bottom plates and growth inhibition was measured using the CellTiter-Glo assay (Promega, Madison, WI, USA). Cytotoxicity curves were measured 24 h, 48 h, and 72 h after drug treatment using a Spectramax M5 plate reader (Molecular Devices, Sunnyvale, CA, USA) with seven colistin concentrations (50, 100, 150, 200, 250, 375 and 500 µM).

### 4.3. Genome-Wide Association and Gene Expression Association with Phenotype

Genotype data were obtained from the 1000 Genomes June 2011 phase I low-pass whole genome SNP genotype release and utilized the GRCh37/hg19. The location and nomenclature of SNPs in the text were updated for the GRCh38/hg38 assembly. Missing values were imputed from the 1000 Genomes Project [61,62,63] as previously described [39]. A list of the cell lines included in each analysis is provided in Supplementary Table S4. Analyses were performed to identify SNPs associated with cellular sensitivity to colistin by regressing the number of minor allele copies for each SNP against log_2_-transformed colistin cytotoxicity IC_50_ values. All SNPs evaluated had a minor allele frequency ≥5% and were in Hardy–Weinberg equilibrium (*p* ≥ 0.001). Regression analyses, including the relationships between colistin IC_50_ with gene or protein expression, and the relationships between genetic variables such as SNPs, baseline gene expression, miRNA, or protein expression were performed in R [64]. Global baseline gene expression levels measured by RNAseq data [38] was used as the metric for mRNA expression. Some expression associations were also evaluated by an Illumina H6 v2 array [42]. Protein expression was measured using microwestern and reverse phase protein arrays as previously published [39]. A *p*-value threshold of ≤0.001 was used in the drug GWAS and in the subsequent analyses for eQTLs and pQTLs.

### 4.4. Expression Quantittative Trait Loci and Protein Quantittative Trait Loci Enrichment Analysis

Top colistin-associated SNPs (*p* ≤ 0.001) were tested for enrichment of eQTLs and pQTLs. A permutation analysis was performed with one thousand random sets of SNPs, generated from the set of HapMap YRI SNPs, each with matching minor allele frequency distributions as the set of significantly associated SNPs. For each random set, the number of eQTLs or pQTLs was determined, yielding a distribution of the expected counts from which an empirical *p*-value for the enrichment was calculated by comparing to the observed count [37]. mRNA-independent pQTLs were examined using regression analysis on protein expression residuals after regression against mRNA levels for the same gene.

### 4.5. Animal Studies and Immunohistochemistry

All animal studies were performed as previously reported [36] and approved by the University of Chicago and Indiana University School of Medicine’s Institutional Animal Care and Use Committee (protocol 10670, approval date 1-24-14). In brief, animals were administered colistin 8 mg/kg twice daily for 3 or 15 days and sacrificed with harvest of their kidneys. Paraffin-embedded sections were stained using an antibody to HOXD10 (200 µg/mL, 1:100 dilution, sc-66926, Santa Cruz, Dallas, TX, USA) according to the manufacturer’s instructions, including the optional antigen retrieval step. Renal staining intensity was scored based on total number of pixels per 40× field (*n* = 4 mice per group, with 10 fields per mouse) as determined by ImageJ 1.49a [65].

### 4.6. Small Interfering RNA Nucleofection

Normal human proximal tubular kidney cells were diluted to 500,000 cells/mL one day prior to nucleofection. Cells were nucleofected using the SF Cell Line Amaxa X-system Nucleofector Kit (Lonza Inc.) and the CA-137 program on Lonza’s Nucleofector Amaxa X-system. Cells were then centrifuged at 90× *g* for 10 min at room temperature and resuspended at a concentration of 1,000,000 cells/20 µL in SF/supplement solution (included in SF Kit Lonza Catalog V4SC2096) and 2000 nM final concentration of All Stars Negative Control siRNA (Qiagen, Inc., Valencia, CA, USA) or a pool of four siRNA constructs for the genes TGIF1, HOXD10 or both mRNAs together (Dharmacon, Lafayette, CO, USA). Cells were allowed to rest for 10 min prior to the addition of pre-warmed (37 °C water bath) REGM media and then for another 5 min in the warm REGM media. Cells were then plated for mRNA harvest or drug treatment. Cells were harvested 24 and 48 h post-nucleofection for gene or protein expression measurements, respectively.

### 4.7. Quantitative Quantitative Real-Time Polymerase Chain Reaction

Quantitative real-time polymerase chain reaction (qRT-PCR) was performed to measure the levels of expression of TGIF1 and HOXD10 in NHPTK cells. A total of 1 million cells were pelleted 24 h after nucleofection, washed in ice-cold PBS, and centrifuged to remove PBS. All pellets were stored at −80 °C until RNA isolation. Total RNA was extracted using the miRNeasy Plus Mini Kit (Qiagen) following the manufacturer’s protocol. Subsequently, mRNA was reverse transcribed to cDNA using the Bio-Rad iScript Reverse Transcription Kit (Bio-Rad, Hercules, CA, USA). The final concentration of cDNA was 25 ng/mL. *GAPDH* was used as an endogenous control using custom made primers (Life Technologies) and iTaq Universal SYBR Green (Bio-Rad) on the Bio-Rad iCycler qRT-PCR system. Primer sequences are given in Table S5. Total reaction was performed in 20 µL volume, which consisted of 10 µL SYBR green, 4 µL cDNA, 0.4 µL of each primer (0.4 µM concentration), and 5.2 µL of water. The thermocycler parameters were 95 °C for 30 s, 40 cycles of 95 °C for 15 s, and then a lower temperature for 30 s (GAPDH: 60 °C, TGIF1: 50 °C, HOXD10: 56 °C), with ramping speeds of 1.6–1.98 °C/s and a melt curve. The C_T_ threshold and baseline for each experiment were set automatically by the Bio-Rad software. 

The ΔΔ*C*_T_ method was used to obtain the relative expression of each gene for samples treated with their associated pool of siRNA or scrambled siRNA. Fold change of the siRNA knockdown as compared to the scramble was determined by the formula fold change = 2^ΔΔ*C*T^. Gene expression for each condition is given as a percentage of expression relative to the scramble control. Each knockdown experiment was conducted three or more separate times with the qRT-PCR samples run in triplicate. Within each cell line, statistical significance was assessed based on an analysis of variance (ANOVA) between time points.

### 4.8. Protein Quantitation by Immunoblot in Normal Human Proximal Tubular Kidney Cells

Western immunoblots were performed to measure the level of protein expression of TGIF1 and HOXD10 in NHPTK cells. Approximately 3 million cells were harvested from a petri dish for each condition (siScramble, siTGIF1, siHOXD10, siTGIF1 + siHOXD10) on five occasions 48 h after nucleofection. Media was removed and cells were rinsed with PBS. Radioimmunoprecipitation assay (RIPA) buffer (0.3 mL) with protease inhibitor was added to the monolayer and cells were removed with a cell scraper 48 h after nucleofection. Cells were centrifuged at 10,000× *g* for 10 minutes at 4 °C and the supernatant was used in subsequent immunoblotting. Protein concentrations of each sample were measured using the bicinchoninic acid (BCA) procedure (Pierce Chemical, Rockford, IL, USA). Samples (20 µg protein) were electrophoresed through a 4%–12% sodium dodecyl sulfate polyacrylamide gel electrophoresis (SDS-PAGE) (Invitrogen Nu-PAGE) under reducing conditions. Proteins were transferred to an Immobilon-P nitrocellulose membrane (Millipore, Bedford, MA, USA) and blocked for 2 h in 5% *w*/*v* milk. Membranes were incubated overnight with rabbit polyclonal anti-HOXD10 (200 µg/mL, 1:200 dilution) in 5% milk. A secondary goat anti-rabbit HRP antibody (sc-2004, 1:2000 dilution) was then incubated for 1 h. A protein molecular size ladder control was run for each membrane with Precision Plus Protein WesternC (Bio-Rad, Hercules, CA, USA). The gel was developed with ECL prime (GE, Piscataway, NJ, USA) and analyzed in a Bio-Rad ChemiDoc MP imaging system. An actin-β control was performed for each membrane with mouse anti-actin-β antibody (sc-47778, 1:250) and goat anti-mouse horseradish peroxidase (HRP; sc-2005, 1:5000). Band density was assessed with Biorad Image Lab 4.1 software and normalized to actin-β for each lane.

### 4.9. Drug Treatment after Nucleofection

Normal human proximal tubular kidney cells were plated in triplicate at a density of 2 × 10^4^ cells/mL in 96-well flat-bottom plates and colistin was added 24 h later. Each knockdown experiment was conducted three or more separate times (at least three biologic replicates and six technical replicates). Cell survival was measured 24, 48, and 72 h post-drug treatment using the Cell-Titer Glo assay (Promega, Madison, WI, USA). Relative change in resistance to colistin was calculated as follows: (Survival_siCondition, colistin (µM)_/Survival_siCondition, control_)/(Survival_siScramble, colistin (µM)_/Survival_siScramble, control_), in which colistin (µM) = colistin treated cells at a given concentration; Control = vehicle treated cells, siCondition = siRNA experimental knockdown group, and siScramble = scrambled siRNA control group.

### 4.10. Small Interfering RNA Statistical Analysis

To assess the size and significance of the effect of siRNA on NHPTK cell survival after colistin treatment, a linear mixed model used was: survival~siRNA + (1|experiment). The mixed-effects model was fit using the *lmer* function from the *lme4* package in R version 2.15.2. Significance of the siRNA term in the model was assessed using a likelihood ratio test.

## Supplementary Materials

Supplementary materials can be found at www.mdpi.com/1422-0067/18/3/661/s1. Figure S1: Histogram illustrating the distribution of cytotoxicity phenotypes in LCLs; Figure S2: Additional association study results; Table S1: SNPs associated with colistin cytotoxicity; Table S2: pQTLs associated with colistin cytotoxicity; Table S3: Associations between colistin cytotoxicity and protein expression; Table S4: LCLs used in each analysis; Table S5: Primer sequences and temperatures.
