# Supplementary material for: Genetic Variants Contributing to Colistin Cytotoxicity: Identification of TGIF1 and HOXD10 Using a Population Genomics Approach
Source: Int J Mol Sci. 2017 Mar 18;18(3):661. doi: 10.3390/ijms18030661 (PMC5372673; doi:10.3390/ijms18030661)
Supplement: Supplementary file 1 [file ijms-18-00661-s001.zip › ijms-181843-supplementary.docx]

Supplementary Materials: Genetic Variants Contributing to Colistin Cytotoxicity: Identification of *TGIF1* and HOXD10 Using a Population Genomics Approach

Michael T. Eadon, Ronald J. Hause, Amy L. Stark, Ying-Hua Cheng, Heather E. Wheeler, Kimberly S. Burgess, Eric A. Benson, Patrick N. Cunningham, Robert L. Bacallao, Pierre C. Dagher, Todd C. Skaar, M. Eileen Dolan


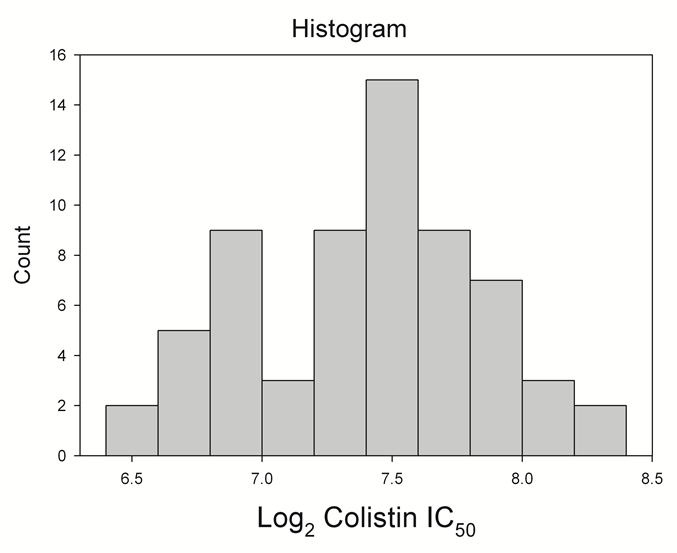


**Figure S1.** Histogram illustrating the distribution of cytotoxicity phenotypes in LCLs.


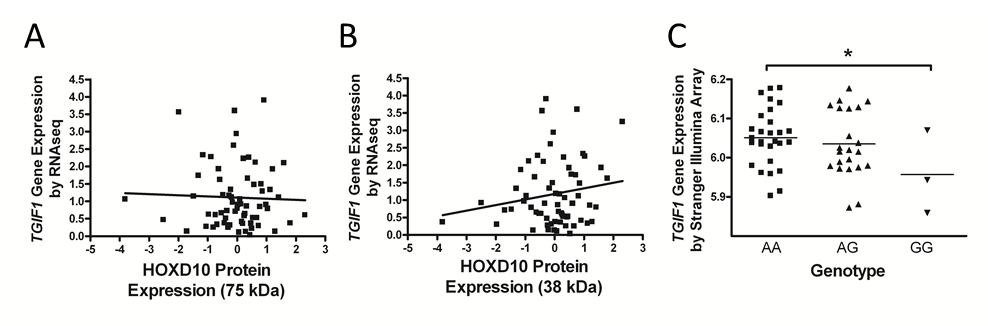


**Figure S2.** Additional association study results: (**A**,**B**) TGIF1 mRNA expression by RNAseq and HOXD10 protein associations were non-significant; and (**C**) *TGIF1* gene expression by Illumina array was lower in the GG genotype group as compared to the AA genotype (* = *p* = 0.05).
